# Consumer-Grade Headphones for Children: Limited Effectiveness of “Level Limiters” When Used With Portable or Home Media Players

**DOI:** 10.1177/2331216519889232

**Published:** 2019-12-23

**Authors:** Michael A. Stone, Mark Harrison, Keith Wilbraham, Melanie Lough

**Affiliations:** 1 Hearing Device Research Centre, Manchester Centre for Audiology and Deafness, School of Health Sciences, University of Manchester, UK; 2 Manchester University Hospitals NHS Foundation Trust, Manchester Academic Health Science Centre, UK; 3 Firecrest Films, Glasgow, UK

**Keywords:** EN50332, WHO safe listening, dynamic range limiting

## Abstract

Consumer-grade headphones for children are frequently packaged or marketed with labels claiming incorporation of an output-level-limiting function. Six pairs of headphones, sold separately from devices with audio interfaces, were selected either from online recommendations or from “best rated” with a large online retailer, the opinions being expressed in 2018 to early 2019. The acoustic outputs in response to an internationally standardized test signal were measured through the ears of a head-and-torso simulator and referenced to equivalent *A*-weighted diffuse-field sound pressure levels. The headphones were tested with a variety of music capable sources found in a domestic environment, such as a mobile phone, tablets, laptop computer, and a home “hi-fi” CD player. To maintain likely homogeneity of the audio interface, the computer-based platforms were manufactured by either Apple™ or certified Android devices. One of the two Bluetooth-linked headphones exhibited level limiting with low distortion (i.e., a compression ratio well in excess of unity). None of the devices wired directly to an audio output performed distortionless level limiting: “limiting” was implemented by a reduction of sensitivity or mechanical limitations, so could be called “soft limiting.” When driven by a laptop or CD player, some were still capable of producing output levels well in excess of “safe-listening” levels of 85 dB(A). Packaging labels were frequently ambiguous and imprecise.

## Introduction

The introduction of workplace legislation in industrialized societies has led to a general reduction in the incidence of noise-induced hearing loss but an increase in recreational exposure (Biassoni et al., 2005; Smith, Davis, Ferguson, & Lutman, 2000). Recreational exposure comes from a variety of sources such as amplified venues and portable/personal music players, which are ubiquitous (annual sales in excess of 1.5 billion devices, International Telecommunications Union [ITU], 2018) and cheap (at least in high- and middle-income countries).

Multiple studies have reported the sampling of sound levels from personal and portable devices such as headphones, “Walkmen,” and MP3 players and found that a modest proportion are exposing their users to sound levels that are potentially injurious (Bradley, Fortnum, & Coles, 1987; Kuras & Findlay, 1974; Shimokura & Soeta, 2012; Twardella et al., 2016; West & Evans, 1990; Williams, 2005). Of increasing concern is the accessibility of these high-sound-level capable devices, especially to a younger audience (ITU, 2018). International standards exist to encourage manufacturers to include safe-listening modes (EN50332-1, 2013; ITU, 2018; World Health Organization [WHO], 2019) and even to incorporate calculation of an accumulated noise exposure in a similar fashion to that used for calculating exposures in an industrial setting. Warnings can then be presented to the user at various exposure-accumulation points.

Calculation of energy doses requires the software to be aware of the power delivered to the headphone as well as the headphone sensitivity. This approach works well as long as the “stock” headphones supplied with the player are in use. Substitution with a headphone of unknown sensitivity renders calculations of accumulated exposure meaningless. The onus then falls on the headphone designer, guided by standards, to select a sensitivity that is suitable for the range of player interfaces with which the headphone is expected to be mated and to provide sufficient technical information on which the user can make an informed choice as to their safety.

The designer’s choice of sensitivity can be guided by international standards, the EN50332 family (EN50332-1, 2013; EN50332-2, 2013), or the WHO (2019) guidelines, which define limits to the sensitivity. EN50332 references a safe level as 85 dB(A) SPL, derogated by committee from the industrial standard of 80 dB(A), over a 40-hr working week (EN60065, 2011, p. 185), the levels being referenced to the diffuse field. The WHO guideline, defined by more recent work, sets two possible maximum output levels, again based on a 40-hr per week rate of exposure, either 80 dB(A) SPL for adults (“Mode 1”) or 75 dB(A) for “sensitive users,” for example, children (“Mode 2”). The cautious approach of “Mode 2” can be justified for two reasons: (a) the smaller acoustic volume in a pediatric ear canal means that levels estimated in standard, adult-referenced, couplers are underestimates (Feigin, Kopun, Stelmachowicz, & Gorga, 1989) and (b) compared with adults, younger listeners have the potential for a greater accumulated lifetime exposure.

Headphones specifically marketed toward children often include promotional statements about the devices being “volume limited” to typically around “85 dB,” implying that they are “safe” or “safer” for use by children. These headphones are often additionally labeled on their use of a very common connector, the 3.5-mm jack plug, making them easily connectible to a variety of media players found in the domestic environment.

The Hearing Device Research Centre at the Manchester Centre for Audiology and Deafness was commissioned by Firecrest Films (Glasgow, UK) to measure the output levels of consumer-grade headphones specifically marketed toward children of a range of ages. All headphones were available as stand-alone, with no companion player. All of the headphones tested came with packaging labels, as well as marketing labels, implying that they incorporated level-limiting technology and should be suitable for a wide range of media players with headphone outputs.

The concept of level-limiting dates back to the early days of radio and telephone (e.g., Wright, 1938) and is understood across many fields of audio engineering to involve some form of level-dependent (i.e., nonlinear) amplification. The timescale on which this nonlinearity operates is a choice made by the circuit designer and ranges from instantaneous (“peak clipping”) to long term (several seconds), as used in automatic gain control. Peak clipping is a relatively cheap solution as it can be implemented by suitable choice of the maximum voltage or current that can be supplied in the amplifier stages. Automatic gain control involves greater cost because of the need for extra components and design sophistication. The purpose of the limiting, however achieved, is to prevent either overload of electrical circuits leading to distortion or overdrive of acoustic transducers, leading to excessive sound levels.

The EN50332 family of standards—specifically EN50332-1 (2013) and EN50332-2 (2013)—is targeted at measuring the headphone output levels when driven by “personal media players.” As defined by EN62368 (2014), p192 “personal music players” are intended for use with headphones or earphones, are battery powered, and are of a size that would fit in a pocket and explicitly exclude mains-powered players. In the context of usage of headphones by a child in the domestic environment, there is a wider variety of possible personal replay devices available. If the limiting technology is effective, then the distinction of size and powering method should be largely immaterial. The aim of the work was therefore to use a generous interpretation of personal media player to include a range of devices likely to be encountered in a domestic environment, such as a laptop and a mains-powered home “hi-fi” CD player. The use of a mains-powered device represents a likely worst case (i.e. the most intense) because the choice of amplifier supply voltages and currents is not so restricted compared with those in a battery-powered design where low-power consumption and hence longer battery life in a light-weight package feature highly in the design choices.

EN50332-2 (2013) concerns the measurement of headphones when supplied separate from the player and provides a measurement method to provide a comparison of the output levels produced. It is this part that formed the structure of the experiment to measure output levels reported here and hence understand the effectiveness of the level-limiting technology of the headphones. Although external amplifiers explicitly targeted at headphones are available, these were not included in the testing as they are commonly marketed with the intention of increasing output levels.

## Method

The six pairs of headphones were identified via recommendations in online articles or as “best buys” with a large online retailer. Sampling was performed in late 2018 to early 2019. Details of the headphones are listed in Table 1, including wording taken from marketing and packaging, as well as the target age range of the children. It should be stressed that, because only one sample of headphone per manufacturer was available, we did not seek to identify “good” or “bad” performers but sought to identify patterns in the manufacture and marketing of such devices. EN50332 does not explicitly require more than one sample of each device to be tested, despite requiring multiple measures after a procedure of place and replace on the test ear. Therefore, for any particular headphone, there is always a possibility that it was a “rogue” device.

**Table 1.** Details of the Six Pairs of Headphones Used in the Testing, Listed in Descending Price Order.

Manufacturer, model name, marketing labels	Price	dB limit and description	Target age and design styling	Connection method to player
Puro Sound BT2200 Headphones “Volume limiter”“Volume governor”	£59.99	85 dB	Children—no ages noted.Most mature design	Bluetooth wireless or wired 3.5 mm
LilGadgets Untangled Pro Headphones	£34.99	93 dB	Children 4+Fairly mature design	Bluetooth wireless or wired 3.5 mm
Kidz Gear Volume Limiting Headphones	£19.98	80 dB–90 dB“Volume limit technology”^a^108 dB without limiter	Ages 2+Middle ground design between childish and more mature	Wired via 3.5 mm with detachable in-line limiter
Snuggly Rascals Penguin Kids’ Headphones	£14.99	85 dB^a^^a^Level may be exceeded under unusual circumstances such as usage of an audio amplifier.	Children 3+Childish design	Wired via 3.5 mm
Peppa Pig“Volume restricted”	£14.99	85 dB	Children 3–7 years oldChildish design	Wired via 3.5 mm
JVC Tinyphones“Volume limiter”	£ 11.99	85 dB/1mW^a^^a^Sound volume may exceed 85 dB depending on use environment.	Children 3+Fairly childish design	Wired via 3.5 mm

*Note.* “dB limit and description” as a column header is deliberately chosen as labeling was often imprecise as to what units were in use.

^a^A caveat from packaging or company website.

### Stimulus

The intention of EN50332-2 is that the headphones should be tested with a random noise signal, called program simulation noise (PSN), which is representative in average level and frequency content of “typical” audio programs. The spectral shape is similar to a pink noise but with the addition of roll-off at low and high frequency. The typical use of dynamic range compression in the recording of many signals in a studio context means that the statistical distribution of sound levels is not the same as that of the original acoustic signal. PSN reflects this reduction. A 30-s sample of PSN, with a spectrum and a small crest factor (6.5 dB), both as defined by HD483.1 S2 (1989), was generated. The low-crest-factor requirement ruled out the use of noise with Gaussian statistics. Therefore, the procedure described in Stone, Moore, and Greenish (2008) was used to generate a “low-noise” (Pumplin, 1985) version. The procedure iterated the timing of the individual spectral components of the noise until the crest factor requirement was met while at the same time also meeting the spectral shape requirements.

To gain more information about the action of any volume limiter from each recording, a composite waveform comprising a variety of test signals was generated in “wav” format, sampled at 44.1 kHz with 16-bit precision. This single format was replayable by all the media players used. Note that conversion to MP3 format would have altered the crest factor of the signal so was not employed.

The time order of the composite signal was as follows:

1. PSN to HD483.1 with a digital root mean square (rms) of −10 dB, 30 s in duration, level, and duration as required by EN50332-1.
2. PSN to HD483.1 with a digital rms of −20 dB, 30 s in duration, and 10 dB lower than that required by EN50332-1.
3. 500-Hz tone pips of 1 s steady duration, with onsets and offsets gated with a 100-ms raised cosine ramp, stepping 3 dB per pip, with digital rms levels of −25, −22, −19, −16, −13, −10, −7, −4, and −1 dB.

All rms values are quoted relative to a full scale sine wave.

The composite test signal was constructed so that more detail could be extracted than required by EN50332-2:

- The two PSN bursts differed in input level so it would be easy to see whether limiting had occurred in the output signal.
- The stepped tone could be used to characterize the limiter characteristic as well as any distortion generated in the process of limiting.
- As the *A*-weighted level correction for 500 Hz is −3 dB, then one step of the tone burst sequence had the same rms as the first burst of PSN.

The intended rms level of EN50332-1 has real-world relevance in that it is close to that used by music libraries and online streaming services, such as Spotify, which now employ “loudness normalization.” The normalization is performed in-house (e.g., Apple’s iTunes Sound Check, Apple, 2019), to defacto standards such as ReplayGain (Robinson, 2002), or to international standards such as ITU 1770 (ITU, 2015; Spotify, 2019) which translate to rms levels of around −13 dB. The stepped tones permitted us to investigate linearity of the transducers, the onset of limiting, if any, for levels exceeding the −10 dB rms of the PSN, as well as the mode of limiting (high fidelity or with distortion, as judged by the generation of harmonics of the test tone).

The translation of the digital level into sound pressure is entirely determined by the output stages of the replay device. EN50332-2 specifies that “Any volume control, tone control or equalisation setting, if any, shall be adjusted to the setting that gives the maximum output.” While all volume controls on test devices were set to maximum, no attempt was made to investigate possible equalization changes such as bass boost, which can occur on some replay devices, as these can vary between manufacturers, adding yet another dimension of variability.

The composite test signal was intended to be replayed from each of four different types of devices:

1. a Samsung Galaxy S7 mobile phone running Android (abbreviated to “Phone”),
2. a Samsung tablet (abbreviated to “Tablet”) and an LG V500 tablet, both running Android,
3. an Apple Macbook Pro 2014 laptop (abbreviated to “Laptop”) and Apple iPad A1673 tablet (abbreviated to “iPad”), and
4. a CD player (Denon DCD 625).

The first three categories described earlier were battery powered and therefore “portable” and “personal,” although the laptop was too big for a pocket. The fourth device, the CD player, was mains powered, making it nonportable. Strictly interpreted, this device falls outside of the intentions of EN50332-2 and the definition of EN62368. However, the provision of a headphone output implies a degree of personal listening is intended. The composite signal was loaded as a digital file to the first three devices and burnt to CD for the fourth.

As the levels recorded from the Apple laptop were higher than from the two styles of Samsung devices (Phone and Tablet), we used the iPad tablet to investigate whether this was a feature of the laptop (not a personal music player, as defined by EN62368), or a more consistent feature of audio interfaces from the same manufacturer. For the two headphones compared with both the iPad and the laptop (JVC Tinyphones and the Peppa Pig products), the near identical levels measured indicated that it was more of an interface feature, at least up to 85 dB(A) SPL output.

The similarity of levels across the Samsung devices again raised a question as to whether this was a manufacturer-imposed limitation or a more generic limitation of an Android-based device. The Android-based LG V500 tablet showed a mean 1.4-dB lower output than the Samsung devices, when delivering via the Puro Sound and the Peppa Pig products. The battery-powered players used for all headphones therefore appeared to be reasonably representative of the Apple and Android families of devices. The iPad and LG V500 will not be considered further beyond reporting of the measures in Table 2.

**Table 2.** Measures of Response to the PSN With Level of −10 dB rms, of Wired Headphone Outputs Into Left and Right Ears of KEMAR Manikin, as (Left Right) Pairs.

Headphone	Media player									
	Phone		Tablet		LG V500	Laptop		iPad	CD player	
Puro Sound	73.0	73.6	72.5	72.3	71.4 70.6	84.8	84.7		79.9	79.4
LilGadgets	87.8	86.6	87.9	87.4		95.6	95.2		97.5	98.7
			*87.4*	*87.7*		*94.9*	*95.1*			
Kidz Gear										
With limiter	76.4	74.9	76.1	74.5		81.2	79.7		88.6	87.0
Without limiter	88.8	86.9	84.2	82.2		92.6	90.8		98.3	96.4
									*97.7*	*96.1*
Snuggly	87.9	84.1	88.3	92.3		95.6	93.9		96.8	93.6
Rascals	*88.4*	*82.5*							*98.3*	*93.1*
Peppa Pig	75.9	76.0	74.8	75.8	72.8 73.9	86.1	86.2	85.2 86.4	85.2	86.6
JVC Tinyphones	79.3	79.1	77.6	78.4		86.6	86.0	85.8 86.6	90.2	91.2
						*86.5*	*86.4*			

*Note.* (*Left right*) pairs (i.e., in italics) indicate measures after removal and replacement of headphones on manikin to maximize outputs. Grayed squares indicate where no measure was performed. Measures are in dB(A) SPL, referenced to the equivalent diffuse field, as required by EN50332-1.

Bluetooth connections to headphones were assessed using three players; the Samsung phone and tablet as well as the Apple laptop.

### Procedure

A head-and-torso simulator (“HATS,” specifically a KEMAR manikin) to IEC60318-7 (2017) with binaural microphones was set up in the middle of a low reverberation ((Reverebration Time) RT_60_ < 120 ms, 0.125–8 kHz), very quiet room (background noise <30 dB(A) SPL). The head was fitted with small silicone-rubber pinnae of low hardness (35 Shore OO hardness). Each meatus was terminated by an EN60318-4 (2010) coupler and a Bruel and Kjaer (Naerum, Denmark) 4192 microphone. The microphone signal was conditioned by a Bruel and Kjaer 2669-L pre-amplifier powered by a GRAS (Holte, Denmark) Type 12AA power module (incorporating a stepped-gain amplifier). The power module signal was fed to a PreSonus (Baton Rouge, LA, USA) V22 SL 2-channel USB soundcard attached to a PC running MATLAB™ (Mathworks, Natick, MA, USA) under Windows™ 7. The power module gain, of either +20 or +40 dB, was chosen so as to ensure use of the full electrical signal range of the soundcard without digital clipping. All recordings were made with a resolution of 24 bits. The microphone sensitivities were calibrated both before and after the recordings using a Bruel and Kjaer 4231 calibrator. No drift in sensitivity had occurred during the recordings.

The headphone under test was placed on the pinnae so that the center of the transducer was as close as practically reasonable to the center of the meatal entry. EN50332-2 specifies five measures of each device with a procedure of place–replace between measures: The intention of the placement is to maximize output level. As we were performing binaural recordings, each measure actually produced two independent readings. With so many combinations of players and headphones to test, for each headphone, the initial placement was adjusted and, using a short-duration test signal, we verified that this achieved maximum output. The headphones were then left mostly undisturbed during the remainder of the testing, but we also verified during the testing of each headphone that at least one place–replace procedure produced no more than a 2-dB difference in measured level. The Snuggly Rascals, being a design incorporating transducers in pockets in a sweatband, were difficult to align consistently with each meatus: The level exhibited large variations with placement. These therefore required more careful positioning and lack of disturbance to ensure a stable measure across players.

The physical format of the limiter for the Kidz Gear was as an extension cable, comprising a 3.5-mm, 3-contact plug to 3.5-mm, three-contact socket, to be fitted in line with the headphone cable. Measures were performed with, and without, this limiter in place.

### Analysis

The meatal recordings of the replayed composite test signal were analyzed by a custom script written in MATLAB which performed the manipulations required by EN50332-2 on the first PSN burst of the composite test signal. These were as follows:

1. Transformation of the frequency response from eardrum to equivalent response in the diffuse field using the response given in IEC60318-7 (2017).
2. Transformation of the frequency response from dB SPL to *A*-weighted dB SPL using the response given in EN61672-1 (2013).
3. Measurement of the total sound power in each 30-s PSN burst from this *A*-weighted diffuse-field spectrum.

Manual measures were performed on the two noise bursts in the composite signal and the tone bursts stepped in level. These measures did not need to be in absolute units. If the difference in level between the two noise bursts was preserved at 10 dB, then there was no evidence of limiting having occurred at the test level required by EN50332. Furthermore, analysis of the tone bursts would show at what level the interstep level change dropped below 3 dB, indicating that some form of limiting was occurring. It should be noted that the stepped-level measurements could exhibit the onset of limiting for several reasons:

- The headphones were exhibiting compression limiting, as understood from a professional audio explanation: A variable gain amplifier, under control of a short-term level measurement circuit, was reducing the signal gain, resulting in low distortion in the controlled signal.
- The player electrical output stage was unable to deliver the drive current to the headphone at such a high level and so was also distorting on a cycle-by-cycle basis.
- The headphone mechanical driver had reached the end of its linear travel and was distorting on a cycle-by-cycle basis

When cycle-by-cycle distortion occurred, it would have required more investigation and equipment to distinguish which of (ii) or (iii) was the primary cause. With the wired headphones, we did not observe any type (i) limiting and hence infer that it was of types (ii) or (iii).

A further subtlety comes in classifying the degree of limiting, when observed. In professional audio applications, this would be defined from the compression ratio, defined as the inverse of the slope of the input–output function for the device under test, when both measures are expressed on a logarithmic scale. Compression ratios exceeding about 10 would be considered as “hard limiting,” as the output level barely changes as a function of input level. Compression ratios less than about 2 would be regarded as “soft limiting.”

## Results

The raw results broken down by headphone and media player are shown in Tables 2 and 3. Table 2 shows the results for wired connections of the headphones. Table 3 shows the results for the wireless connections of the headphones. Repeat measures are shown in italics for some combinations of headphone and media player. They typically show differences of less than 1 dB, with worst-case difference being 1.6 dB with the Snuggly Rascals headphones. As previously noted, it was difficult to achieve a consistent alignment with the meatus for maximum output.

**Table 3.** Measures of Response to the PSN With Level of −10 dB rms, of Bluetooth-Connected Headphones Into Left and Right Ears of KEMAR Manikin, as (Left Right) Pairs.

Headphone	Media player					
	Phone		Tablet		Laptop	
Puro Sound	78.8^a^	79.2^a^	79.1^a^	79.2^a^	79.6^a^	79.8^a^
					*78.8* ^a^	*78.9* ^a^
LilGadgets	86.7	87.4	84.3	86.5	86.4	86.2
			*86.1*	*87.2*		

*Note.* Measures are dB(A) SPL. (*Left right*) pairs (i.e., in italics) indicate measures after removal and replacement of headphones on manikin to maximize outputs.

^a^Hard-limiting active.

**Figure 1.**
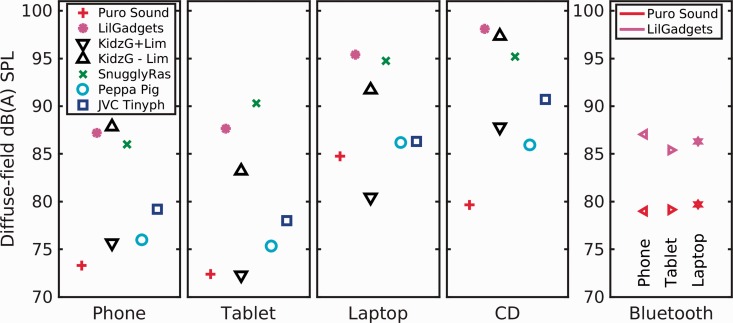
Output levels as referenced to EN50332-2 for the headphones according to player type (left-hand four panels). For two headphone pairs, the output levels when driven in Bluetooth mode are shown in the far right-hand panel, for each of the Bluetooth capable sources.

The results from Tables 2 and 3 are summarized as plots in Figure 1. The left-hand four panels of Figure 1 show the results for the headphones in wired mode, sorted across panels by the media player used and sorted within panels according to the listing order in Table 1. The right-hand panel shows the results for the two headphones capable of operating via a Bluetooth link. All the results represent the average of the left-ear and right-ear recordings (excluding the repeat measures). For the Kidz Gear headphones, two results are presented, one for when the in-line limiter was in use (downward black arrows) and one for when the limiter was bypassed (upward arrows). The figure has been scaled so that the EN50332 “safe level” of 85 dB(A) is in the middle of the ordinate. Compliant devices should therefore be recognized as lying close to or below this level.

Generally, the Android devices resulted in the lowest outputs. Allowing for a 3-dB margin of measurement error, only two devices were on the edge of exceeding the 85-dB(A) limit of EN50332. Although the LilGadgets headphone levels exceeded 85 dB(A), this was below the manufacturer’s limiting level of “93 dB” stated on the packaging. For the laptop and CD player, the outputs are typically around 8.5 and 10 dB higher, respectively, averaged over all devices used with their limiters. The in-line limiter provided with the Kidz Gear headphones produced a mean 11-dB reduction in output level across all devices (standard deviation = 1.0 dB), entirely by a linear process.

**Figure 2.**
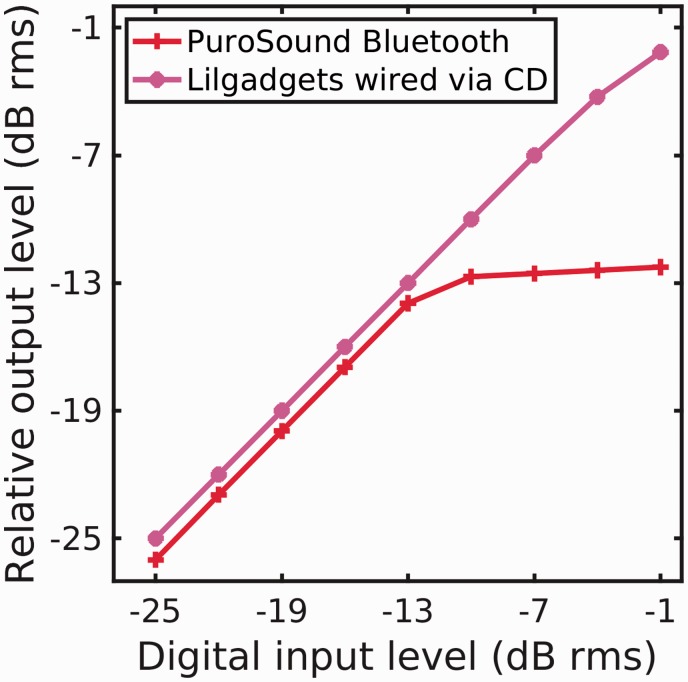
Growth function of relative output level as a function of digital input level of a 500-Hz tone. The traces have been offset for clarity. The Puro Sound provided distortion-free hard limiting for the last four steps of the input sequence. The LilGadgets showed soft limiting and marked harmonic distortion for the highest two input levels.

The only instance of distortion-free compression limiting was seen was in the case of the Puro Sound via its Bluetooth link. Figure 2 shows the input–output function for this headphone and link combination in response to the stepped 500-Hz tones of the test signal (crosses, red trace). A slope of unity would indicate linear behavior. The last four steps, with slope less than unity, show the limiting in action. The compression ratio (inverse of the slope of the input–output function) was 20:1, a hard limiter. Distorted limiting was seen most commonly with the CD player as the source. An example of this is shown in Figure 2 (asterisks, magenta trace) for the LilGadgets headphones, again in response to the stepped 500-Hz tone. Analysis of the frequency content (not shown) and level change indicated that the last two steps introduced distorting soft limiting. We did not investigate whether this was a limitation of the CD player or the headphones.

For the laptop and CD player, much higher output levels were seen. Although it can be argued that the latter is not a battery-powered device, and that the laptop may be less intentioned as a “personal music player” (although we found no evidence that it produced levels any different from a tablet from the same manufacturer), these levels are disturbing. Several of the headphones were marketed with labels of “volume limiting,” “volume governor,” and “volume limited” (see Table 1, first column), which may lead to the consumer assuming that the headphones would be safe when used with these sources.

As can also be seen in Table 1, the labels commonly seen with these products are imprecise: *e.g.* “85 dB” has no units, “85dB/mW” is a sensitivity measure, not a limit. Some headphones came with warnings, that, under certain conditions of use, levels could exceed 85 dB and even approach “108 dB” without the limiter.

## Conclusions

Although there are many headphones which claim a level-limiting function, are targeted at a pediatric audience, and are deemed “safe” when tested strictly in accordance with EN50332-2, combinations of the devices with some common domestic appliances are capable of producing potentially injurious levels.

The following can be concluded from our sampling:

1. The labeling on the packaging was often technically imprecise, while marketing labels used in retail outlets could be even more imprecise.
2. The maximum output levels depended strongly on what sort of domestic device the headphones was plugged in to.
3. It was the rare exception that hard limiting was observed (the distortion-free hard limiting of the Puro Sound BT2200 via Bluetooth). Otherwise, any manufacturer’s claim of “limiting” was primarily achieved by a linear process: a deliberate design decision of setting the headphone sensitivity to be low. Under such a configuration, a hard limit was not possible. Any soft limiting then observed was as either distortion from the media player amplifier stages or a mechanical limitation of the transducer.

We suggest that parental supervision is still necessary, even when using devices fitted with “volume limiting technology.” Caveat emptor (buyer beware).

## Authors’ Note

Firecrest Films (author M. H.) commissioned the work and selected the headphones and the media players. Technical design, measures, and interpretations were not performed “for hire” but performed solely by the University of Manchester employees (M. A. S., K. W., and M. L.), with no editorial influence from Firecrest Films other than for legal purposes, which produced no changes.
